# Fatal spirorchiidosis in European pond turtles (*Emys orbicularis*) in Switzerland

**DOI:** 10.1016/j.ijppaw.2022.01.004

**Published:** 2022-01-11

**Authors:** Katja Schönbächler, Philipp Olias, Olivia K. Richard, Francesco C. Origgi, Eva Dervas, Stefan Hoby, Walter Basso, Inês Berenguer Veiga

**Affiliations:** a Berne Animal Park, Tierparkweg 1, 3005, Bern, Switzerland; b Institute of Animal Pathology, Department of Infectious Diseases and Pathobiology, Vetsuisse Faculty, University of Bern, Länggassstrasse 122, 3012, Bern, Switzerland; c Institute of Veterinary Pathology, Vetsuisse Faculty, University of Zurich, Winterthurerstrasse 268, 8057, Zurich, Switzerland; d Institute of Parasitology, Department of Infectious Diseases and Pathobiology, Vetsuisse Faculty, University of Bern, Länggassstrasse 122, 3012, Bern, Switzerland

**Keywords:** Spirorchiidosis, Freshwater turtle, *Emys orbicularis*, *Spirorchis* sp., Conservation, Invasive species

## Abstract

Infections with intravascular digenean trematodes of the Spirorchiidae family (spirorchiidoses) are of great conservation concern both in marine and freshwater turtles due to their pathogenic potential. Between 2014 and 2021, *Spirorchis* sp. infections associated with granulomatous inflammation and sudden death were detected in European pond turtles (*Emys orbicularis*) from three conservation breeding facilities in Switzerland. Blood fluke eggs associated with lesions were found in the intestine, spleen, testis, skeletal musculature, heart, kidneys, stomach, pancreas, liver, lung, and meninges from nine pond turtles submitted for necropsy and in the intestinal content from five of these animals. Two novel polymerase chain reactions (PCRs) targeting the *28S ribosomal RNA* gene and the ITS2 region and subsequent sequencing revealed 100% nucleotide identity with a *Spirorchis* sp. previously isolated from an Escambia map turtle (*Graptemys ernsti*) in the USA. Our findings suggest a spill-over event secondary to direct or indirect contact with invasive North American turtle species in Switzerland. We describe the clinical, haematological, ultrasonographical, endoscopical, parasitological, pathological, and molecular findings associated with spirorchiid blood fluke infections of the *Spirorchis* genus in *E. orbicularis*, as well as the biosecurity measures that were developed to prevent the spread of this parasite among breeding and highly endangered free-ranging *E. orbicularis* populations in Switzerland.

## Highlights

• Spirorchiidosis was recorded in three Swiss *Emys orbicularis* breeding facilities.
• *Spirorchis* sp*.* infection was detected by parasitological and molecular methods.
• Spill-over events from invasive American turtle species are suspected.
• Biosecurity measures are essential to avoid *Spirorchis* sp. spread into wild populations.

## Graphical abstract

**Figure:**
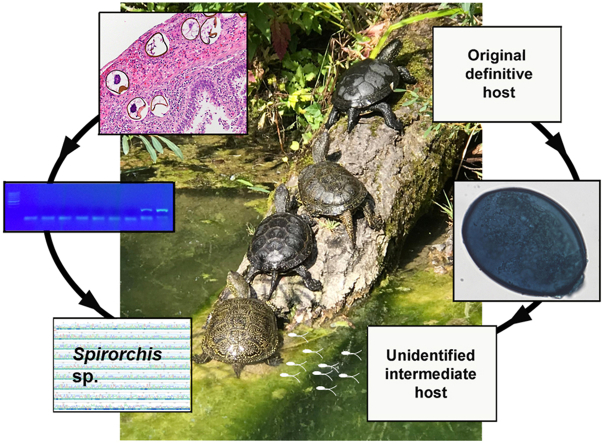

## 1 Introduction

Infections with intravascular digenean trematodes of the Spirorchiidae family (spirorchiidoses) can be associated with clinical disease and are of great conservation concern both in marine and freshwater turtles due to their impact on endangered and vulnerable species worldwide (Chapman et al., 2017, 2019; Iglesias et al., 2015). Vermetid, pulmonated, prosobranch and fissurelid gastropods (Corner et al., 2021; Cribb et al., 2017; Holliman et al., 1971; Pieper, 1953; Stacy et al., 2010; Turner and Corkum, 1977; Wall, 1940, 1941), as well as polychaete worms (de Buron et al., 2018) have been found to act as intermediate hosts for different species of marine and freshwater spirorchiid blood flukes. The infection of the definitive hosts is thought to occur following direct skin penetration by fork-tailed cercariae (Chapman et al., 2019; Holliman et al., 1971). Adult flukes are most commonly found in the aorta and cardiac chambers (Chapman et al., 2017; Gordon et al., 1998; Holliman and Fisher, 1968; Santoro et al., 2007; Stacy et al., 2010), even if other locations including the mesenteric (Roberts et al., 2019), hepatic (Stacy et al., 2010), and central nervous system vasculature (Gordon et al., 1998; Holliman et al., 1971; Jacobson et al., 2006; Stacy et al., 2010) have been described in some species. The presence of the adult parasites may lead to arteritis, endarteritis, and thrombosis (Chapman et al., 2017; Flint et al., 2010; Gordon et al., 1998; Santoro et al., 2007; Stacy et al., 2010; Werneck et al., 2016). Furthermore, egg release into the bloodstream is associated with widespread granulomatous inflammation, which can affect any organ (Chapman et al., 2017; Flint et al., 2010; Gordon et al., 1998; Santoro et al., 2007; Stacy et al., 2010; Werneck et al., 2016), but their presence is most often described in the central nervous system, the lungs, and the gastrointestinal tract (Chapman et al., 2017; Stacy et al., 2010). The eggs reaching the gastrointestinal lumen are released with the faeces into the environment, but other excretion routes such as expectoration, death and decomposition of hosts have also been described (Chapman et al., 2019). Clinical signs associated with spirorchiidoses are non-specific and include lethargy, anorexia, emaciation, neurological signs, and sudden death (Flint et al., 2010; Holliman and Fisher, 1968; Holliman et al., 1971; Jacobson et al., 2006; Johnson et al., 1998; Stacy et al., 2010; Work et al., 2015).

While the occurrence of spirorchiidoses has been extensively documented in several marine species such as green (*Chelonya mydas*) (Chapman et al., 2017; Flint et al., 2010, 2015) and loggerhead sea turtles (*Caretta caretta*) (Jacobson et al., 2006; Stacy et al., 2010), infections with spirorchiid blood flukes are only occasionally reported in freshwater turtles and concern mostly *Spirorchis* spp. in North American Emydidae such as painted turtles (*Chrysemys picta*) (Holliman and Fisher, 1968; Holliman et al., 1971; Johnson et al., 1998; Snyder, 2004), red-eared sliders (*Trachemys scripta elegans*) (Johnson et al., 1998), map turtles (*Graptemys* spp*.*) (Roberts et al., 2019), Eastern box turtles (*Terrapene carolina*) (Yonkers et al., 2015), and river cooters (*Pseudemys concinna*) (Roberts et al., 2018).

European pond turtles (*Emys orbicularis*) are omnivorous turtles (Çiçek and Ayaz, 2011) from the Emydidae family whose habitat spans from East and Central Europe to regions rimming the Mediterranean Sea (Fritz, 2001, 2003). They are classified as “near threatened” according to the International Union for Conservation of Nature (IUCN) Red List (Ducotterd et al., 2020), whereas the Swiss population is classified as critically endangered (Monney and Meyer, 2005). This species is known to host the spirorchiid blood fluke *Spirhapalum polesianum*, which has been identified in healthy and diseased *E. orbicularis* specimens from Poland, Russia, Ukraine, Germany, and Romania (Ejsmont, 1927; Jennemann and Bidmon, 2009; Mihalca et al., 2007; Sharpilo, 1960; Snyder, 2004). In 2015, a spirorchiidosis outbreak was reported in free-ranging *E. orbicularis* from Galicia in Spain (Iglesias et al., 2015). Morphological analysis of adult parasites recovered from the heart of diseased animals revealed *Spirorchis elegans*, a spirorchiid blood fluke that has been found in the vascular system of several North American Emydidae turtles, and whose type host is *T. scripta elegans* (Iglesias et al., 2015; Platt, 1992) as causative agent. Since this invasive turtle species is known to be free-ranging in Spain, and considering that all known members of the *Spirorchis* genus were identified in turtles originally from the United States, this outbreak was considered secondary to a spill-over event following the introduction of invasive turtle species into the wild (Iglesias et al., 2015). However, molecular evidence supporting this assumption was lacking.

In Switzerland, a breeding program was developed to preserve *E. orbicularis* and support its reintroduction into the original habitat. Between 2014 and 2021, several cases of infection with spirorchiid blood flukes associated with clinical disease and mortality were detected in three *E. orbicularis* breeding facilities in this country. Here we report the clinical, haematological, ultrasonographical, endoscopic, pathological, parasitological, and molecular findings associated with spirorchiid blood fluke infection in *E. orbicularis* from Switzerland and describe the containment measures taken to prevent the spread of this parasite among breeding and free-ranging *E. orbicularis* populations.

## 2 Material and methods

Ethics approval. All procedures were approved by the cantonal committee for animal experimentation and are in accordance with the Swiss animal welfare legislation (license number BE 31/19).

Study population. All performed clinical activities were carried out within the scope of a broad health assessment of captive and free-living *E. orbicularis* populations in Switzerland between 2019 and 2021 (Schönbächler et al., 2022). The *E. orbicularis* specimens that were included in this study were captive individuals from three *E. orbicularis* breeding facilities belonging to the SwissEmys Group, an association founded in 2012 in Switzerland, whose main aim is to reintroduce *E. orbicularis* in suitable habitats in Switzerland. Affected breeding facilities were based in the cantons of Solothurn (A), Freiburg (B), and St. Gallen (C). In total, infections with spirorchiid blood flukes were diagnosed in nine (ID1 – ID9; three animals from breeder A, two from breeder B, and four from breeder C) out of the 31 *E. orbicularis* specimens that were submitted for histopathological analysis to the Institutes of Animal Pathology of the University of Bern and Veterinary Pathology of the University of Zurich between 2010 and 2021 (Table 1). These were six female, two male, and a turtle of unknown sex aged one to 11 years. Four animals (ID1 - ID3 and ID5) were found dead and originated from groups in which an increased mortality was detected, but unfortunately no additional casualties were available for *post-mortem* investigation. One of these animals (ID2) had displayed pond escape and locomotion impairment prior to death. The remaining five turtles were humanely euthanized. These animals displayed unspecific clinical signs, namely pond escape (ID4), severe apathy (ID1, ID4, ID6 - ID9), and anorexia (ID4, ID8 and ID9). Euthanasia was performed by injecting 100 mg/kg body weight (BW) of pentobarbital i.v. (Esconarkon, Streuli, 8730 Uznach, Switzerland) under deep anaesthesia (20–30 mg/kg ketamine [Ketasol 100, Graeub, 3018 Bern, Switzerland] combined with 0.2–0.3 mg/kg medetomidine i.m. [Dorbene, Graeub, 3018 Bern, Switzerland]). Particularly, two of these animals (ID6 and ID7) belonged to a group of four turtles from breeder C and were included among those selected to be released into the wild after undergoing a complete clinical and virological examination and a first deworming with fenbendazole (Panacur, MSD, 6006 Luzern, Switzerland, 50 mg/kg p.o.). However, ID6, ID7, and a third animal from the group displayed a sudden, severe debilitation one day after deworming with fenbendazole. Therefore, it was decided that all animals of the group ought to be euthanized and submitted for necropsy. The remaining three animals that were euthanized (ID4 and ID9 from breeder A and ID8 from breeder C) presented severe apathy and anorexia and were submitted for intensive care and medical treatment to the quarantine facilities of Berne Animal Park for one day to 3 weeks. Particularly, ID8 received a treatment against trematodes using praziquantel (Caniquantel, Graeub, 3018 Bern, Switzerland, 25 mg/kg, q3h x 3, s.c.) during this period.

**Table 1:** Pathological and parasitological findings of the nine European pond turtles (*E. orbicularis*) specimens infected with spirorchiid blood flukes that were submitted for pathological examination at the Institute for Animal Pathology, University of Bern (n = 8) and at the Institute of Veterinary Pathology, University of Zurich (n = 1, ID1).

ID No.	Breeder	Necropsy	Sex	Age	Organs displaying Spirorchiidae egg-related lesions	Coproscopy (Sedimentation technique)	Molecular analysis (FFPE tissue)^a^	Molecular analysis (eggs)^b^
1	A	June 2014	unknown	6 years	Intestine, spleen kidney	Not performed	PCR +	Not performed
2	B	April 2017	f	5 years	Intestine, skeletal musculature, kidney, pancreas, liver, lung, spleen and meninges	Not performed	PCR +	Not performed
3	B	September 2018	f	1 year	Lung, liver, spleen	Not performed	PCR +	Not performed
4	C	May 2019	f	11 years	Intestine, kidney, skeletal muscle	Not performed	PCR +	Not performed
5	A	August 2019	m	Adult	Intestine, spleen, testis	Spirorchiidae eggs	PCR +	Not performed
6	C	July 2020	f	unknown (4 years?)	Intestine, spleen, pancreas	Spirorchiidae eggs, *Spiroxys contortus* eggs, coccidial oocysts	PCR +	PCR +
7	C	July 2020	f	4 years	Intestine, lung, kidney, stomach, spleen, pancreas	Spirorchiidae eggs, *Spiroxys contortus* eggs	PCR +	PCR +
8	C	July 2020	f	6 years	Intestine, kidney, stomach, spleen	Spirorchiidae eggs; *Serpinema* adult parasites	PCR +	PCR +
9	A	May 2021	m	9 years	Intestine, stomach, testis, spleen, pancreas, heart, liver, kidney, tongue	Spirorchiidae eggs	PCR +	PCR- (only few eggs present)

a All sequenced *28S rRNA* and ITS2 amplicons were identical among themselves and were deposited in GenBank (accession numbers OL412668-OL412676 and OM154169-OM154177, respectively).

b *28S rRNA* amplicons obtained from faecal eggs shared the same sequence observed in FFPE tissues; f = female; m = male.

Ultrasonographical analysis. This analysis was performed on animals ID4 and ID9 while housed at the Berne Animal Park. A portable unit (MyLabDeltaVet Esaote, Trezzano sul Naviglio, 20090, Italy) with associated micro convex transducer (SC3123: 4–9 MHz) was used. The two cervicobrachial and prefemoral windows were scanned. The ultrasonographic study was reviewed using an open-source Digital Imaging and Communications in Medicine (DICOM) commercial viewing software (Horos v3.3.6).

Endoscopical analysis. This was performed on ID8 and ID9 while housed at the Berne Animal Park under general anaesthesia with medetomidine and ketamine (see above), and meloxicam (Metacam, Boehringer, 4002 Basel, Switzerland, 0.2 mg/kg s.c.). Access to the coelomic cavity was achieved via the left prefemoral fossa with the animal in right lateral position. The skin was aseptically prepared, and a 3.0 mm skin incision was made. The underlying musculature and peritoneum were perforated with a blunt trocar sleeve. A 4.0 mm x 18-cm 30° telescope (Karl Storz, 78532 Tuttlingen, Germany) connected to an endoscopic video unit (Tele Pack Vet X 20045020, Karl Storz) was used. Carbon dioxide (CO_2_) was introduced via the valve of the trocar sleeve with a maximum pressure of the mechanical insufflator of 5–8 mmHg (Endo-Arthroflator-VET 62432520, Karl Storz).

Haematological and biochemical blood analysis. Blood was collected from the dorsal coccygeal vein (*V. coccygealis dorsalis*) from ID4, ID8 and ID9 while housed at the Berne Animal Park. It was immediately transferred to a 0.5 ml Li-Heparin tube (Sarstedt, Sevelen, 9475, Switzerland). The maximum amount of blood collected did not exceed 0.4% of body weight. Blood chemistry was evaluated with a VetScan VS2 (Abaxis Europe, Griesheim, 64347, Germany) using the avian/reptilian rotor (ID4, ID8) and at the Clinical Laboratory, Vetsuisse Faculty, University of Zurich (ID9), respectively.

Pathology. Full necropsy and histopathological examination of the nine affected turtles were performed. Samples from the liver, lung, and kidneys, as well as intestinal content were collected for bacteriological, virological, and parasitological analysis. A detailed description of the performed virological analysis is provided elsewhere (Schönbächler et al., 2022). In addition, a set of internal organs (liver, spleen, reproductive tract, urinary bladder, lung, heart, brain, skeletal muscle, skin, and gastrointestinal tract, etc.) were sampled from each individual and immediately fixed in 10% neutral buffered formalin. Tissues were processed, embedded in paraffin, cut at 4 μm, and stained with haematoxylin and eosin (H&E) according to standard protocols.

Coproscopical analysis. Samples of intestinal content from cases ID5 - ID9 were obtained after necropsy and processed by the sedimentation-zinc chloride flotation (s.d. 1.35) and sedimentation techniques (Deplazes et al., 2016).

Molecular analysis. To confirm the microscopical diagnoses and to obtain information about the genetic background of the spirochiid parasites involved in these cases, a molecular characterization based on the amplification and sequencing of a 255 bp fragment of the highly variable D3 domain of the *28S ribosomal RNA* gene and of a 315 bp fragment of the internal transcribed spacer 2 (ITS2) region of the Spirorchiidae family was performed. DNA was extracted from formalin-fixed paraffin-embedded (FFPE) tissues from ID1 - ID9 in which trematode eggs had been observed, as previously described (Müller et al., 2015). Additionally, DNA was extracted from trematode eggs recovered from faecal sediments (ID5 - ID9) and from cloacal swabs (ID6 and ID7) collected prior to euthanasia using a commercial DNA extraction kit (NucleoSpin DNA Stool Mini kit, Macherey-Nagel, Germany). The PCR reaction for the amplification of the *28S rRNA* gene was performed using the newly designed primer Spiro F-1 (5′ TTGAAACACGGACCAAGGAGTTTAAC 3′) and Spiro R-1 (5′ TTCACCATCTTTCGGGTCTCAAC 3′), while the amplification of the ITS2 sequence was performed by using the newly designed primer ITS2-Spirofw1 (5′CTTGGATGTGTGCCAGCTGG 3’) and ITS2-Spirorev2 (5′CACGTCTGATCCGAGGTCAGG 3′). Both PCR reactions were performed with the QIAGEN Multiplex Master Mix and Promega GoTaq polymerase, respectively, using the following thermocycling conditions: 94 °C 15 min–35 x (94 °C/45 s, 55 °C/45 s, 72 °C/45 s) – 72 °C 10 min–4 °C (∞; for the *28S rRNA* gene); 95 °C 2 min–40 x (95 °C/40 s, 62 °C/40 s, 72 °C/30 s) – 72 °C 5 min–4 °C (∞; for the ITS2 region). The obtained PCR products were resolved by electrophoresis on 2% agarose gels stained with ethidium bromide, purified with a commercial kit (DNA Clean & Concentrator-5 Zymo Research, USA), and subsequently Sanger sequenced in both directions (Microsynth, Balgach, Switzerland) with the same primers used for amplification. The obtained sequences were compared with those available in GenBank using the BLAST tool (http://blast.ncbi.nlm.nih.gov/Blast.cgi) and deposited in GenBank. The amplified partial *28S rRNA* and ITS2 sequences, identical sequences available within NCBI, as well as sequences from related parasites of the Spirorchiidae family were aligned with MUSCLE within the Geneious prime software. GenBank accession numbers are given in Fig. 3. Sequences of the *28S rRNA* gene were trimmed to 206 bp and used to construct a Maximum Likelihood phylogenetic tree in IQ-TREE (Trifinopoulos et al., 2016) with the TMP3+G substitution model as determined by jModelTest 2.1.10 (Darriba et al., 2012). *Alaria alata* was used as an outgroup. For the aligned and trimmed 274 bp of the ITS2 region, SplitsTree v4.17 (Huson and Bryant, 2006) was used to calculate an unrooted phylogenetic network using the neighbour-net method and the F81 model determined by jModelTest.

Preliminary intermediate host screening. Approximately 30 snail specimens from the *Radix*, *Lymnaea* and *Planorbarius* genera originating from affected ponds from breeders B and C were collected and dissected under a stereomicroscope (Leica MS5, Leica Microsystems, Wetzlar, Germany) to check for infection with spirorchiid stages.

## 3 Results

Nine turtles (ID1− ID9) displaying lesions consistent with spirorchiid blood fluke infection were investigated (Table 1). Haematological and blood chemistry analysis were performed on ID4, ID8 and ID9 (only biochemistry) prior to euthanasia. ID4 and ID9 revealed increased aspartate aminotransferase, and creatine kinase (CK). Additionally, ID4 presented elevated uric acid levels and decreased phosphorus (P) levels in comparison with reference levels (Suppl. Tables 1 and 2, adapted from Schönbächler et al., 2022). ID8 displayed a relative monocytosis and lymphopenia associated with elevated CK and decreased P levels. Ultrasonographical examination of ID4 revealed an increased amount of coelomic fluid, which raised the suspicion of coelomitis, while ID9 displayed multifocal hyperechoic areas in the liver and segmentally thickened intestinal walls. Additionally, the endoscopic examination of ID9 revealed the presence of a moderate amount of a clear, reddish effusion in the coelomic cavity, pale liver with multifocal dark patterns, diffuse dark kidneys, and multifocal slightly red areas in the subserosa of the testis.

**Fig. 1:**
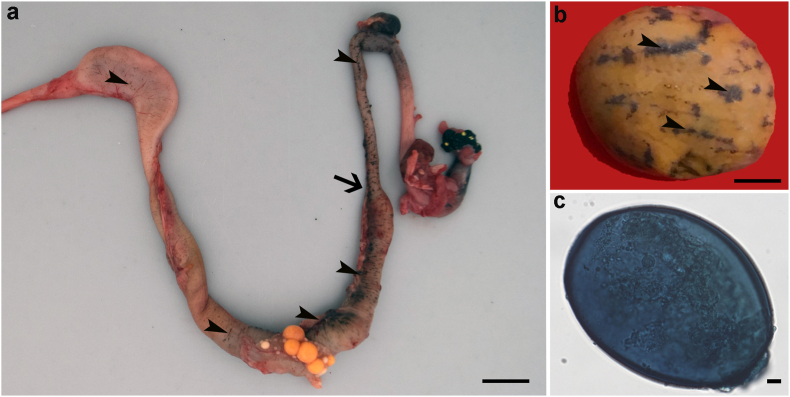
Gross findings and parasitology, (a) Gastrointestinal tract from a 6-year-old female European pond turtle (*Emys orbicularis*, ID8) displaying large numbers of spirorchiid eggs in the subserosal vessels (arrowheads), which are more visible in the intestine. Note the focal stricture of the intestine (arrow) with proximal severe dilation. This section was filled with a large amount of necrotic material. Bar 1 cm. (b) Autolytic testis from ID5 displaying similar lesions to the ones observed in the gastrointestinal tract from ID8 (arrowheads). Bar 25 mm. (c) Aspect of a spirorchiid egg stained with methylene blue identified following sedimentation from intestinal content. Light optical microscope, Bar 10 μm. (For interpretation of the references to colour in this figure legend, the reader is referred to the Web version of this article.)

Gross necropsy findings were highly variable, except for emaciation, which was observed in all animals but ID6 and ID7. Some carcasses displayed solely intestinal (ID1) or widespread petechial haemorrhages (ID2 and ID3). In other cases, myriads of black round elements (<0.5 mm in diameter) arranged in minimally elevated, up to 1 cm short lines, which extended over the gastric and intestinal serosa (ID4, ID5 and ID8; Fig. 1a) and testis (ID5; Fig. 1b) were observed. Particularly, ID4 and ID8 displayed a segmental thickening of the intestinal wall, with proximal dilation and intraluminal accumulation of abundant necrotic material (ID8; Fig. 1a) and deposition of fibrinous material in the serosal surfaces. Additionally, ID6 and ID7 had a diffusely swollen, pale liver along with a moderate gastric infestation with the nematodes *Serpinema* sp*.* and *Spyroxis* sp*.* These adult nematodes were identified morphologically according to Martínez-Silvestre et al., (2015) and Demkowska-Kutrzepa et al. (2021).

**Fig. 2:**
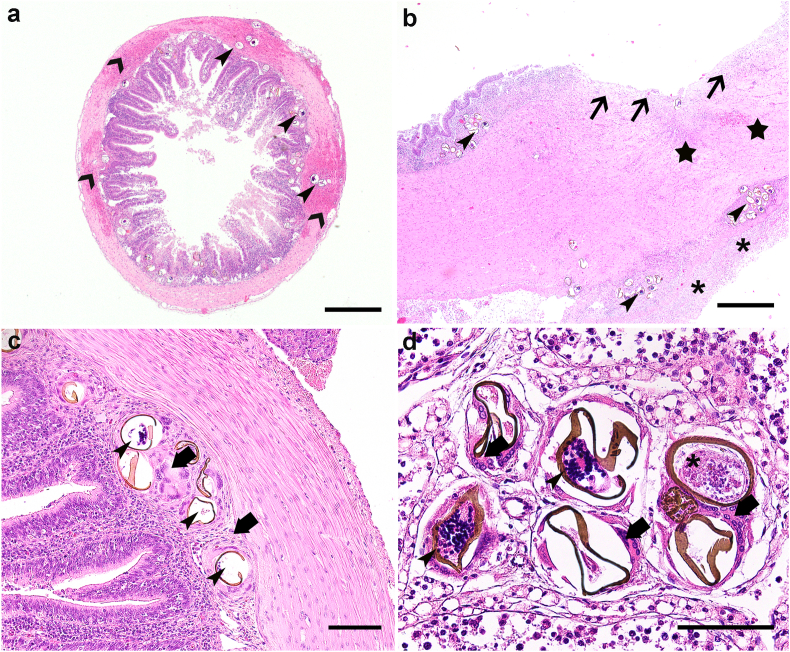
Histopathological findings, (a) Five-year-old female European pond turtle (*Emys orbicularis*, ID2), small intestine. Multiple intravascular trematode eggs (narrow arrowheads) are present in the tunica muscularis, and submucosa associated with severe granulomatous inflammation and acute haemorrhage (large arrowheads). H&E staining, bar 500 μm. (b) Eleven-year-old female European pond turtle (*Emys orbicularis*, ID4), large intestine. The mucosa displays a focal deep ulceration (arrows) with replacement of the underlying submucosa and tunica muscularis by fibrous tissue (stars) and severe granulomatous coelomitis (asterisks). Multiple trematode eggs are present intravascularly, particularly in the subserosal vasculature (arrowheads). H&E staining, bar 200 μm. (c) ID2, small intestine. Focal granulomatous reaction with multinucleated giant cells (arrows) displaying intracytoplasmic, partially disrupted trematode eggs (arrowheads). H&E staining, bar 100 μm. (d) Adult male European pond turtle (*Emys orbicularis* ID5), testis. Interstitial granulomatous reaction composed of multinucleated giant cells (arrows) displaying intracytoplasmic embryonated (arrowheads) and non-embryonated (asterisk) trematode eggs. H&E staining, bar 50 μm.

Histologically, most animals (ID1, ID2, ID4, ID5 and ID8) displayed a severe necrotizing, ulcerative and granulomatous enterocolitis (Table 1) with a large amount of intralesional, intravascular trematode eggs measuring approximately 100 × 45 μm with a 3 μm thick, brown, refractile shell (Fig. 2a and c). These eggs were often fragmented and observed in the cytoplasm of multinucleated giant cells, with surrounding acute haemorrhages and heterophilic inflammation, along with frequent thrombosis of the intestinal and mesenterial vasculature. Lesions compatible with severe coelomitis were also observed in cases ID4 (Fig. 2b) and ID8, and a necrotizing hepatitis with intralesional bacterial colonies, which was consistent with a septicaemic spread, was observed in ID4. Additionally, in all cases we observed a widespread, mild to severe granulomatous inflammation associated with similar trematode eggs in several organs, particularly in the spleen, testis (Fig. 2d), skeletal musculature, heart, kidneys, stomach, pancreas, liver, lung, and meninges (Table 1). No adult blood flukes were found in any of the cases. Additionally, ID6, ID7, and a co-housed animal (not included in this study) displayed severe hepatic changes such as severe, diffuse hydropic hepatic degeneration and lipidosis, and a mild to moderate ulcerative and lymphocytic gastritis at the sites of attachment of the above-mentioned gastric nematodes identified upon necropsy. Lastly, ID9 displayed lesions consistent with a bacterial orchitis.

**Fig. 3:**
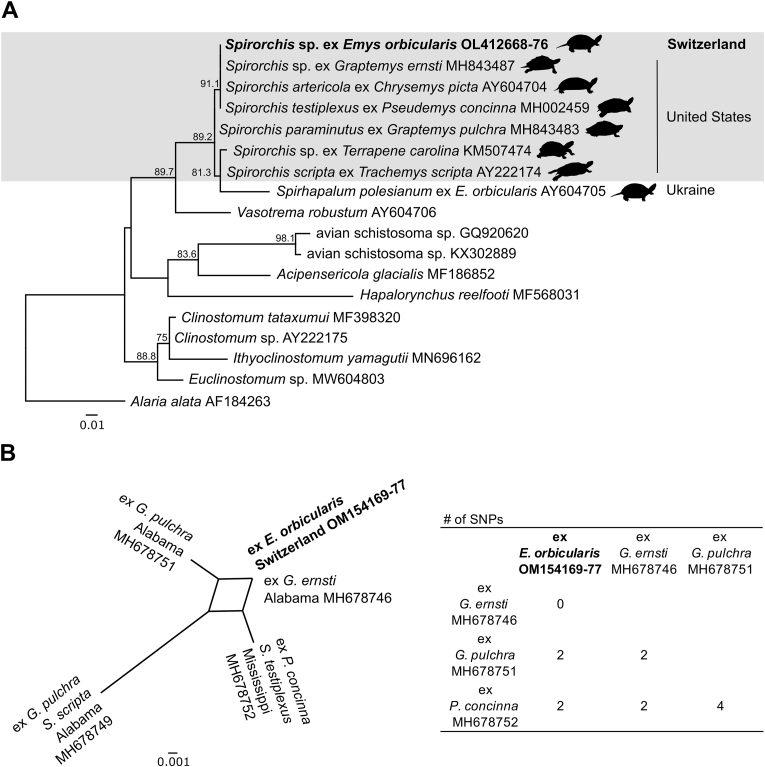
Phylogenetic analysis, (a) Maximum likelihood phylogenetic tree of 206 bp of the *28S rRNA* gene of members of the family Spirorchiidae, with *Alaria alata* as outgroup. Members of the *Spirorchis* genus are boxed. Parasite names are provided, followed by host names (top clade only), GenBank accession numbers and country of parasite discovery. Bootstrap values above 70 are shown, and branch lengths corresponding to the number of base substitutions are indicated by the scale bar. (b) Unrooted phylogenetic network of 274 bp of the ITS2 region of *Spirorchis* spp. recently described from North Amercian turtle species (Roberts et al., 2019) and Swiss *Emys orbicularis*. Host names, location of discovery and GenBank accession numbers are given. Note that the unnamed *Spirorchis* parasite described from *Graptemys ernsti* (AL, United States) is 100% identical in both the partial *28S rRNA* (MH843487), and ITS2 (MH678746) sequences amplified from all Swiss turtle specimens.

In cases ID5 - ID9, trematode operculated eggs measuring 113.4 μm (144 - 82) x 80.8 μm (55–106) could be detected by sedimentation from the intestinal content collected upon necropsy (Fig. 1c). Additionally, positive PCR results for trematodes of the Spirorchiidae family were obtained from all FFPE tissue samples (ID1 - ID9) and from trematode eggs recovered from ID5 - ID9, while cloacal swab samples from ID6 and ID7 yielded negative PCR results.

All sequenced amplicons of the *28S rRNA* gene and the ITS2 region obtained from FFPE tissues were identical among them and were deposited in GenBank (accession numbers OL412668 - OL412676 and OM154169-OM154177, respectively). 28S *rRNA* gene amplicons with the same sequence were also obtained from faecal eggs recovered from some of the turtles (ID6 - ID8).

The amplified 28S *rRNA* sequences shared 100% identity (206/206 bp) with sequences of *Spirorchis* spp. recovered from *P. concinna* in the Pascagoula River, MS, USA (GenBank MH002459.1, MH843489.1) (Roberts et al., 2018, 2019), *Graptemys* *ernsti* in the Yellow River, Alabama, USA (GenBank MH843487.1) (Roberts et al., 2019), and *Spirorchis artericola* from *C. picta* in the Reelfoot Lake, Lake County, Tennessee, USA (GenBank AY604704.1) (Snyder, 2004). A phylogenetic analysis placed the *Spirorchis* sp. identified in the pond turtles in a large clade with closely related parasites all belonging to *Spirochis* spp. identified in North American freshwater turtles from the Emydidiae family (Fig. 3A). The genetic difference between the sister taxons *S. artericola* (1,265 bp), *S. testiplexus* (1,615 bp) and *Spirorchis* sp. ex *G*. *ernsti* (1,579 bp) is negligible (<0.3%, Supplement. Table 3) and absent within the variable D3 domain when compared to the shorter amplicons sequenced in this study. The amplified partial ITS2 sequences were all 100% identical to the innominate *Spirorchis* sp. specimen recovered from *G. ernsti* in the Yellow River, AL, USA (GenBank MH678746; Roberts et al., 2019; cp. Fig. 3A), and different in at least 2 nucleotide positions to all other previously published sequences (Fig. 3B). This result supported our suspicion that a *Spirorchis* sp. of North American origin was the etiologic agent behind all nine infections detected in *E. orbicularis* in Switzerland.

Following identification of *Spirorchis* sp*.* in these breeding facilities, collection, and dissection of approximately 30 snail specimens from the *Radix*, *Lymnaea* and *Planorbarius* genera originating from affected ponds from breeders B and C was performed under a stereomicroscope. However, no *Spirorchis* stages could be identified in any of these specimens, and no molecular analysis was performed.

## 4 Discussion

This is the first report of an infection by a *Spirorchis* sp. in *E. orbicularis* turtles in Switzerland, as well as the first molecular characterisation of a *Spirorchis* sp. infecting freshwater turtles in Europe. A systemic granulomatous inflammation of variable severity associated with the presence of spirorchiid eggs, which was particularly striking in the intestine, could be observed in all affected turtles. These lesions were the most likely cause of the severe clinical signs or death in the turtles. Notably, although some of the cases included in this study had an epidemic nature, others were sporadic. For example, ID6 and ID7 belonged to a group of four turtles that were raised together. However, spirorchiid eggs were identified neither histologically nor in the coprological analysis of the remaining two animals. Considering that a third turtle of this group displayed similar clinical signs and hepatic changes, and that the parasitic lesions displayed by ID6 and ID7 were rather mild, the most likely explanation is that the sudden apathy displayed by these three animals was triggered by deworming with fenbendazole and not by the parasite-related lesions, even if this drug is commonly used in chelonians at even higher dosages without side effects (Galosi et al., 2021; Giannetto et al., 2007). This case indicates that *Spirorchis* sp. infection in *E. orbicularis* may not always be associated with severe and obvious clinical disease, particularly at the early stages of infection.

The diagnosis of spirorchiidoses *intra vitam* remains challenging due to the lack of robust *ante-mortem* diagnostic techniques. Therefore, it is very difficult to enrol only confirmed spirorchiid-free *E. orbicularis* specimens for breeding. Several coprological, serological and molecular testing strategies were applied in marine turtles (Chapman et al., 2019). Particularly, studies in loggerhead turtles showed that copromicroscopy may be an adequate technique to diagnose spirorchiid blood fluke infections, even if it does not allow an accurate quantification of the parasite burden (Marchiori et al., 2018), and faeces collection from turtles is often cumbersome. In the current study, trematode eggs could be identified occasionally in the faeces of *E. orbicularis* from the affected breeding facilities by sedimentation technique and in the intestinal content of five turtles submitted for analysis. Unfortunately, no serological test is currently available, and one limitation for the development of this technique is the lack of an adequate antigen source since it is difficult to obtain enough eggs or adult parasites from affected animals.

Additionally, although ultrasonography and endoscopy in ID4 and ID9 allowed the detection of lesions consistent with coelomitis prior to death, these methods lack the necessary resolution to detect specific lesions associated with *Spirorchis* sp. infection. Also, we were unable to recover adult parasites upon necropsy in any of the affected specimens, even though the presence of *Spirorchis* spp. has been previously described in the heart, the brain, spinal cord, gut, spleen, and lungs of experimentally infected *C. picta picta* (Holliman et al., 1971), in the heart of *E. orbicularis* (Iglesias et al., 2015), and in the heart, mesenteric vasculature, oesophagus, liver, lung, intestine, bladder, and trachea of turtles of the *Graptemys* genus (Roberts et al., 2019). Therefore, in most cases we were able to diagnose an infection by histopathology, and the parasite identity by two newly established 28S *rRNA* and ITS2 PCRs from FFPE tissues containing trematode eggs. These PCR protocols also allowed the molecular identification of *Spirorchis* spp*.* eggs recovered following sedimentation of the intestinal content from some of the affected animals. Although we are aware that the consensus primer PCRs used in our study might not be able to detect multiple spirorchiid species simultaneously present in a specimen, we did not find any evidence for coinfections (such as double peaks) in all chromatograms analysed.

In all cases presented in this study, it was not possible to elucidate how the *E. orbicularis* turtles became infected. The molecular results suggest a close relationship between the spirorchiid flukes infecting Swiss *E. orbicularis* and *Spirorchis* species detected in Emydidae turtles in the USA, which may also explain why *Spirorchis* infection in *E. orbicularis*, a European member of the *Emydidae* family, is productive. This close relationship could be explained (i) by a spill-over event from invasive turtle species, as it was previously reported in Spain (Iglesias et al., 2015), or (ii) by the natural occurrence of autochthonous so far undetected *Spirorchis* species in Switzerland. Notably, putative authochthonous *Spirochis* spp. would have to be molecularly identical in the relatively variable D3 domain of the *28S rRNA* and ITS2 sequences amplified in this study to *Spirorchis* species in the USA. To the best of our knowledge, infections caused by *Spirhapalum polesianum*, the spirorchiid blood fluke that was previously described in the literature from *E. orbicularis* in Europe (Ejsmont, 1927; Jennemann and Bidmon, 2009; Mihalca et al., 2007; Sharpilo, 1960; Snyder, 2004) were never recorded in Switzerland. Apart from the cases described in this study, the only additional case of spirorchidiidoses at the Pathology Institutes of the Vetsuisse Faculties from Bern and Zurich since 2010 was in a stripped mud turtle (*Kinosternon baurii*) that was housed at the Berne Animal Park and which was submitted for necropsy in 2013. However, this animal was never in contact with *E. orbicularis* specimens, and the spirorchiid blood fluke that was identified in this case using the same PCR protocol for the *28S rRNA* gene described in this manuscript was *Hapalorhynchus reelfooti* (unpublished data). None of the affected breeders housed non-native turtle species in the same pond with the affected *E. orbicularis*, although one breeder had kept two *C. picta bellii* in the same water circuit of the affected ponds. Since many of the *E. orbicularis* animals from the SwissEmys breeders came from rescue centers and private collections, it is likely that these animals had previous contact with American turtle species such as *T. scripta scripta* and *Graptemys* sp., which are well represented among the turtles that have been imported to Europe as pets and released to the wild (Kopecky et al., 2013; Rzezutka et al., 2020). However, the successful transmission of this parasite between autochthonous and invasive turtle species implies the existence of compatible intermediate hosts in the environment of the infected turtles, which may also have circulated between ponds in the absence of a common water circuit. Little is known about spirorchiid life cycles in both marine and freshwater turtles and respective intermediate hosts. Some authors believe that there may be a low prevalence of infected intermediate hosts in the wild (Chapman et al., 2019; Holliman et al., 1971). However, overinfestation and water contamination in captivity are likely associated with a significant increase in the prevalence of infected intermediate hosts. Particularly, a prevalence of 37% could be detected in vermetid gastropods infected with *Amphiorchis* sp. cercariae in a Spanish aquarium containing infected loggerhead turtles (Cribb et al., 2017). Identified intermediate hosts for the *Spirorchis* genus described in the literature to date include several species of pulmonated gastropods of the *Helisoma* genus (Holliman and Fisher, 1968; Pieper, 1953; Wall, 1941), which can also be found in Europe. In our case, no parasite stages could be detected in *Radix*, *Lymnaea* and *Planorbarius* snails collected from affected ponds from breeders B and C following the diagnosis of spirorchiidosis in those facilities. However, the sample size was very small and, consequently, these results would need to be revaluated in the context of a broader study.

Following the identification of *Spirorchis* sp*.* as the causative etiologic agent in three affected conservation breeding facilities, containment measures were developed for Swiss *E. orbicularis* official breeders to avoid the spread of this parasite among the breeding *E. orbicularis* populations, as well as from the breeding facilities to free-ranging turtles. These measures include the following key points: (i) animals from affected ponds cannot be released into the wild, exchanged with other breeders, or transferred to new facilities; (ii) snail introduction into turtle ponds should be avoided, and those already present should be reduced as much as possible; (iii) breeding facilities should keep only one turtle species at a time to avoid interspecies transmission of pathogens; (iv) and *E. orbicularis* (other than hatchlings without pond contact) must be submitted to a thorough health check and prophylactically treated against *Spirorchis* sp. prior to reintroduction in the wild. Praziquantel has been used for spirorchiidoses treatment in marine turtles (Adnyana et al., 1997; Stacy et al., 2017) and has been shown to be effective at controlling adult flukes (Adnyana et al., 1997), even if it is unlikely to be effective in the cases where extensive thromboembolism caused by intravascular eggs is present (Innis et al., 2017). Following the pharmacokinetic studies performed in loggerhead turtles (Jacobson et al., 2003), the currently recommended protocol for the SwissEmys breeders, including the animals that are introduced in the breeding facilities, is the treatment with praziquantel (25 mg/kg q 3h x 3, p.o.). However, further studies are needed to determine the efficacy of this drug as well as other deworming drugs for the treatment of spirorchiid blood flukes in *E. orbicularis*. Additionally, a more thorough survey of parasites from non-native turtle species is paramount to better understand how the affected *E. orbicularis* became infected by *Spirorchis* sp. parasites.

## 5 Conclusions

This study reports the occurrence of fatal *Spirorchis* sp. infections in *E. orbicularis* in Switzerland, which might have been introduced by invasive North American turtle species. Besides developing eradication and control programs against invasive turtle species in the wild, further research is needed to identify potential intermediate hosts of these parasites, and to develop robust *ante-mortem* diagnostic tools and effective treatment protocols adapted to *E. orbicularis*.

## Funding

This research did not receive any specific grant from funding agencies in the public, commercial, or not-for-profit sectors and was supported by internal funding.

## Declarations of competing interest

The authors declare that they have no known competing financial interests or personal relationships that could have appeared to influence the work reported in this paper.
