# Supplementary material for: Fatal spirorchiidosis in European pond turtles (Emys orbicularis) in Switzerland
Source: Int J Parasitol Parasites Wildl. 2022 Jan 11;17:144–51. doi: 10.1016/j.ijppaw.2022.01.004 (PMC8777241; doi:10.1016/j.ijppaw.2022.01.004)
Supplement: Multimedia component 1 [file mmc1.docx]

**Supplementary Tables:**

**Supplementary Table 1:** Hematology reference intervals (RI) of *E. orbicularis* without detectable clinical signs from Switzerland.

| Parameter | n^a^ | RI | Mean | Median | SD | 90% LCI | 90%UCI |
| --- | --- | --- | --- | --- | --- | --- | --- |
| Hematocrit % | 115 | 3.6-24.6 | 14.3 | 14 | 5.3 | 2.5-5 | 23.1-26.1 |
| Corr. Leucocyte count  (*10E3/ul) | 82 | 0.57-8.47 | 3.37 | 3.3 | 1.72 | 0.28-0.83 | 6.11-9.07 |
| Heterophils relative (%) | 76 | 8.96-47.69 | 25.57 | 25 | 9.43 | 8.5-11.43 | 45.11-50 |
| Eosinophils relative (%) | 76 | 0.91-40.25 | 13.84 | 12 | 9.79 | 0.41-1.8 | 34.56-47.01 |
| Basophiles relative (%) | 76 | 4.19-33.8 | 18.99 | 19.25 | 7.37 | 2.16-6.48 | 31.34-36.29 |
| Monocytes relative (%) | 76 | 0.5-9.08 | 4.16 | 4 | 2.07 | 0.5-1.5 | 8.04-10 |
| Lymphocytes relative (%) | 76 | 12.89-68.41 | 37.19 | 36 | 13.25 | 11.5-19 | 63-73.5 |
| Heterophils (*10E3/μl) | 76 | 0.13-2.19 | 0.86 | 0.78 | 0.53 | 0.09-0.21 | 1.88-2.55 |
| Eosinophils (*10E3/μl) | 76 | 0.03-1.55 | 0.44 | 0.35 | 0.38 | 0.02-0.05 | 1.25-1.9 |
| Basophiles (*10E3/μl) | 76 | 0.08-1.55 | 0.62 | 0.58 | 0.37 | 0.04-0.15 | 1.35-1.77 |
| Monocytes (*10E3/μl) | 76 | 0.01-0.44 | 0.15 | 0.12 | 0.12 | 0.007-0.014 | 0.34-0.73 |
| Lymphocytes (*10E3/μl) | 76 | 0.11-4.04 | 1.33 | 1.06 | 0.99 | 0.08-0.24 | 3.19-6.03 |

^a^ n = 115 for hematocrit includes 30 males, 51 females, and 34 individuals with undetermined sex (either juvenile or inconclusive sex determination); n = 82 for corrected leukocyte count includes 24 males, 30 females, and 28 individuals with undetermined sex; n = 76 for the remaining parameters includes 24 males, 27 females, and 25 individuals with undetermined sex.

SD = Standard Deviation; LCI = Lower Confidence Interval; UCI = Upper Confidence Interval

**Supplementary Table 2:** Blood chemistry reference intervals (RI) of *E. orbicularis* without detectable clinical signs from Switzerland assessed with the VetScan VS2.

| Parameter^a^ | n^b^ | RI | Mean | Median | SD | 90% LCI | 90% UCI |
| --- | --- | --- | --- | --- | --- | --- | --- |
| AST (U/L) | 41 | 38.4-248 | 102.5 | 90 | 46.3 | 38-46.8 | 181.9-249 |
| CK (U/L) | 39 | - | 414.2 | 339 | 245 | - | - |
| UA (μmol/L) | 31 | - | 42.5 | 34 | 38.3 | - | - |
| Glu (mmol/L) | 41 | 1.9-9.81 | 4.74 | 4.6 | 1.96 | 1.63-2.33 | 8.31-11.44 |
| tCa (mmol/L) | 40 | 2.09-4.23 | 2.73 | 2.58 | 0.5 | 2.03-2.18 | 3.53-5.69 |
| Phos (mmol/L) | 41 | 0.53-2.19 | 0.95 | 0.86 | 0.31 | 0.53-0.59 | 1.47-2.22 |
| TP (g/L) | 41 | 15.2-72.2 | 33.2 | 32 | 11.2 | 15 - 20.2 | 56.3-73 |
| Alb (g/L) | 41 | 3-21 | 10 | 9 | 4.2 | 3-4.1 | 17.8-21 |
| Glob (g/L) | 18 | - | 25.6 | 20 | 12.6 | - | - |
| K (mmol/L) | 40 | 2.9-7.88 | 5.42 | 5.15 | 1.18 | 2.43-3.37 | 7.14-8.36 |
| Na (mmol/L) | 41 | 115.4-145 | 135.8 | 137 | 6.3 | 115-125 | 144.8-145 |

^a^AST = aspartate aminotransferase, CK = creatine kinase, UA = uric acid, Glu = Glucose, tCa = total Calcium, Phos = phosphate, TP = total protein, Alb = albumin, Glob = globulin, K = potassium, Na = sodium

^b^ n = 40 for tCa and K includes 14 males, 12 females, and 14 individuals with undetermined sex (either juvenile or inconclusive sex determination); n = 41 for AST, Glu, Phos, TP and NA includes 14 males, 13 females, and 14 individuals with undetermined sex; n = 39 for CK includes 14 males, 11 females, and 14 individuals with undetermined sex; n = 31 for UA includes 12 males, 10 females, and 9 individuals with undetermined sex; n = 18 for Glob includes 6 males, 4 females, and 8 individuals with undetermined sex.

SD = Standard Deviation; LCI = Lower Confidence Interval; UCI = Upper Confidence Interval

**Supplementary Table 3:** Partial *28S rRNA* percent sequence identity of sister taxons *S. testiplexus*, *S. artericola*, and *Spirorchis* sp. ex *Graptemys ernsti*.

|  | ***S. testiplexus* MH002459** | ***S. artericola* AY604704** | ***Spirorchis* sp. ex *G. ernsti* MH843487** |
| --- | --- | --- | --- |
| **MH002459** |  | 99.76 | 99.68 |
| **AY604704** | 99.76 |  | 99.76 |
| **MH843487** | 99.68 | 99.76 |  |
